# Patient education as a foundation for effective COPD care

**DOI:** 10.1017/S1463423625100522

**Published:** 2025-11-10

**Authors:** Barbara Gonçalves, Audrey Cund, Caroline Sime, Eileen Harkess-Murphy, Joanne Lusher

**Affiliations:** 1 School of Allied and Community Health, London South Bank University, London, UK; 2 Faculty of Medicine and Dentistry, Queen Mary University of London, London, UK; 3 School of Health and Life Sciences, University of the West of Scotland, Ayr, UK; 4 Scottish Partnership for Palliative Care, Edinburgh, UK; 5 School of Health and Life Sciences, University of the West of Scotland, Paisley, UK; 6 Regent’s University London, London, UK

## Introduction

Chronic diseases, such as chronic obstructive pulmonary disease (COPD), are among the leading causes of morbidity and mortality worldwide, posing significant challenges for both patients and healthcare systems (GBD Chronic Respiratory Disease Collaborators, 2020). Unlike acute conditions, which often require immediate and short-term treatments, chronic diseases demand long-term management and ongoing care (Ferrara *et al.*, 2022). As a result, people with chronic conditions may need to be actively involved in their own care to ensure optimal outcomes (Krist *et al*., 2017). This reality exposes the importance of empowering patients to take an active role in their care, marking a shift from traditional, paternalistic models to patient-centred approaches which prioritize education, self-management, and shared decision-making.

According to the World Health Organization (WHO), primary health care is a “whole-of-society approach” which not only integrates health services but also aims to empower individuals, families, and communities to take an active role in managing their own health (WHO, 2018). This global instruction to strengthen patient agency provides an essential context for reconsidering how education and engagement are structured within chronic disease management.

## Evolving role of patient education and engagement in COPD management

The traditional medical model of the doctor-patient relationship presents a situation in which a patient comes to an expert, a medical practitioner, for a consultation and supplementation of their knowledge and understanding (Parsons, 1951). Many traditional care models remain rooted in a paternalistic approach, often overlooking the patient’s own expertise, preferences, and capacity for self-management (Harris *et al*., 2008; Roberts *et al*., 2018). However, this tends to be poorly suited for patients with chronic diseases, who often have a great level of expertise and information (Kelly, 2015).

The limitations of paternalistic models become starkly evident when viewed through the lens of the WHO’s definition, which highlights empowerment as a foundation for equitable and people-centred care. In this context, COPD education which relies on hierarchical relationships can undermine patients’ capacity for self-management and limit their involvement in decision-making. Nevertheless, encouraging progress has been made in recent years, with the landscape of COPD management shifting and moving beyond a paternalistic model towards one that values patient education, engagement, and partnership. A growing body of evidence recognized that effective self-management of COPD is not achieved through information delivery alone, but through active collaboration, rapport-building, and shared decision-making between patients and healthcare providers (Benzo *et al*., 2022; Blackstock *et al*., 2018). Without this foundation, even well-intentioned education efforts may fall short, particularly when psychosocial challenges go unacknowledged.

Accordingly, the WHO stresses that empowerment ought to move decisively beyond the paternalistic model of merely instructing patients. Instead, it requires creating the conditions for individuals to set their own health priorities, co-develop their care plans and have a real say in decisions about their treatment. This reorientation challenges entrenched hierarchical power dynamics in healthcare and represents a key step to improving adherence, resilience, and lasting health gains.

## Gaps in patient education and the role of nurses

Low health literacy is connected with lower treatment adherence; therefore, proper education of people with COPD and their carers is crucial as it may facilitate their engagement in care, resulting in improved coping strategies and treatment adherence (Gonçalves *et al*., 2025a; Muellers *et al*., 2019; Muijsenberg *et al*., 2024). Nevertheless, patient education remains uneven in both content and delivery. While areas such as smoking cessation, medication use, and exercise are often well covered, psychosocial concerns, such as stress, fatigue, depression, social life, and end-of-life planning are frequently overlooked (Siltanen *et al*., 2020). Therefore, the psychosocial aspects of patients’ education need to be addressed more thoroughly.

The way people with COPD perceive their disease helps them adjust self-management in order to improve illness outcomes (Kaptein *et al*., 2014). Effective communication and understanding of the diagnosis are essential for patient engagement (Kwame and Petrucka, 2021; Roberts *et al*., 2008, Sharkiya, 2023). Thus, the quality of information provided to the patients is vital. Importantly, the type of healthcare professional involved in patient education influences both the focus and quality of information provided. Siltanen *et al*. (2020) showed that specialist medical workers provided education more systematically compared to primary care professionals. Physicians and nurses covered different areas of education, with doctors emphasizing the diagnostic procedures and treatment of COPD and nurses focusing on practical management of COPD such as inhalation technique, mouth care, and nutrition. Nurses, especially in primary care, were more active than doctors regarding psychosocial aspects of education, such as well-being, fatigue, stress, anxiety, and depression management. However, despite their importance, support from community nurses is often reported as insufficient, with patients noting infrequent visits and identifying this as an area in need of improvement. Furthermore, a survey of COPD specialist nurses in the community, found that there is little service delivery and a mismatch between the effectiveness of the service and its provision (Candy *et al*., 2007). Therefore, given the critical role of nurses in providing holistic education and psychosocial support, these services seem to require restructuring in order to be more available to the patients and meet their needs.

## Role of dialogue in fostering self-management

In chronic disease management, active engagement can bolster self-management skills and improved quality of life (Johnson *et al*., 2016). People with COPD who lack clean information about their prognosis may hold unrealistic beliefs or misunderstand the seriousness of their condition, undermining their ability to plan, cope, and self-manage (Gauronskaite *et al*., 2016). Conversely, in a study with people with COPD, those who received clear communication from their pulmonologists and nurses exhibited a strong understanding of their condition and treatment, allowing them to feel more in control of their care, whereas those who perceived a lack of dialogue and information from healthcare professionals struggled with managing their treatment effectively (Gonçalves *et al*., 2025b). Hence, constructive collaboration with healthcare providers may help improve patients’ empowerment, build their confidence, and support their sense of autonomy.

Yet, even among engaged patients, success is not guaranteed. Bucknall *et al*. (2012) revealed that certain demographics, particularly older adults living alone, struggled more with self-management. This suggests the need for tailored interventions and enhanced support for vulnerable subgroups. While frameworks for patient activation exist, there is limited evidence on how best to tailor COPD self-management strategies to different populations (Yadav *et al.*, 2018).

## Conclusion

This editorial urges researchers, clinicians, and policymakers to rethink the goals and methods of COPD care. Despite evidence that structured patient involvement improves outcomes, current systems often fail to equip patients with the understanding, confidence or emotional tools to participate fully in their care. The traditional doctor-patient dynamic, though valuable in certain contexts, may not be sufficient for the needs of chronic disease patients. A shift towards patient-centred care, where patients are educated about their condition and actively engaged in their care, is crucial for improving self-management and health outcomes in COPD. Healthcare professionals, particularly nurses, play a vital role in providing holistic, comprehensive education that addresses both medical and psychosocial aspects of COPD care. Healthcare systems ought to invest in training professionals to engage in meaningful dialogue, expand the role of community nurses and embed psychosocial education into routine practice. Therefore, transforming COPD management requires more than incremental adjustments; it calls for realignment with standards such as the WHO’s (2018) vision, which places empowerment and partnership at the heart of primary health care. Transcending paternalistic information-giving, healthcare systems and professionals are encouraged to adopt approaches which actively involve people with COPD as true partners in their care. By embracing patient-centred models and recognizing the full spectrum of patients’ needs, we can advance towards more effective, compassionate, and sustainable chronic disease management.

## Data Availability

No new data were generated or analysed in support of this publication.
